# Risk factors, mortality and acute kidney injury outcomes in cirrhotic patients in the emergency department

**DOI:** 10.1186/s12882-018-1061-8

**Published:** 2018-10-20

**Authors:** Paulo Ricardo Gessolo Lins, Wallace Stwart Carvalho Padilha, Carolina Frade Magalhaes Giradin Pimentel, Marcelo Costa Batista, Aécio Flávio Teixeira de Gois

**Affiliations:** 1 Discipline of Nephrology, Federal University of São Paulo, Rua Botucatu, 591 - 15 ° andar - Cj153 - Vila Clementino, São Paulo, SP 04023-062 Brazil; 2 Discipline of Medicine of Urgency and Evidence-Based Medicine from the Department of Medicine, Federal University of São Paulo, Rua Napoleão de Barros, 865 - Vila Clementino, São Paulo, SP 04023-090 Brazil

**Keywords:** Liver cirrhosis, Acute kidney injury, Hospital mortality, KDIGO, Progression of AKI

## Abstract

**Background:**

Acute kidney injury (AKI) is common in cirrhotic patients and is associated with negative outcomes. The aim of this study was to evaluate the presence of AKI and its progression according to KDIGO (Kidney Disease: Improving Global Outcomes) criteria in cirrhotic patients admitted to the emergency department and to determine the association of AKI with hospital mortality.

**Methods:**

This retrospective study included 258 cirrhotic patients admitted to the emergency department of a university hospital from March 2015 to February 2017. AKI was diagnosed and classified according to the KDIGO criteria.

**Results:**

The overall incidence of AKI in cirrhotic patients was 53.9%, and the overall hospital mortality was 28.4%. Mortality was associated with the presence, stage, and progression of AKI. Patients with AKI stage 1 and sCr < 1.5 mg/dl (KDIGO 1a) had a lower mortality rate than patients with AKI stage 1 and sCr > 1.5 mg/dl (KDIGO 1b). In the logistic regression analysis, three variables were independently associated with hospital mortality: cancer, AKI and progression of AKI.

**Conclusions:**

According to the data presented, a single measure of creatinine is not enough, and there is a need for meticulous follow-up of the renal function of patients with hepatic cirrhosis hospitalized in an emergency unit. In addition, this study reinforces the need for subclassification of KDIGO 1 in cirrhotic patients, since patients with acute renal injury and creatinine greater than 1.5 mg/dL present a worse clinical outcome.

## Background

Acute kidney injury (AKI) is a common complication in patients with liver cirrhosis who are admitted to the emergency department, and it is related to significantly higher mortality rates among this population [1–8]. AKI pathogenesis in cirrhotic patients is intimately related to hemodynamic changes secondary to liver failure and to a self-perpetuating process that ultimately leads to renal and splenic vasoconstriction, promoting decoupling between renal supply and demand and ultimately promoting AKI [9].

Over time, different definitions of AKI have been proposed for cirrhotic patients [10]. In 2012, a universal definition of acute renal injury was proposed by the KDIGO group [11]. However, although this definition is widely applied in different populations, there are few validation studies of KDIGO in cirrhotic patients [2, 12], and such studies rarely consider the context of the emergency department [4].

In addition, an attempt to better describe and identify cirrhotic patients with AKI, is to implement a substratification of KDIGO stage 1 into 1a and 1b, with a creatinine value of 1.50 mg/dL as the discriminatory threshold [7]. Such stratification proposes that those patients belonging to subgroup 1a would present a short-term mortality similar to the patients in the non-AKI group, which is different from those in subgroup 1b, who would present a higher odds ratio for mortality when compared to those in the non-AKI control group [2]. Another important tool is to evaluate the progression of AKI since patients who do not show improvement or stabilization of renal function will have a worse prognosis [13].

This study aimed to characterize a population of cirrhotic patients treated at the emergency department based on risk factors associated with worse prognosis for mortality and development of AKI. We also applied the KDIGO criteria for AKI within the first 7 days of hospitalization, testing the possible correlation between mortality and substratification of the KDIGO 1 group in stages 1a and 1b, and finally, looking for an association between progression of AKI with mortality in this population.

## Methods

**Fig. 1:**
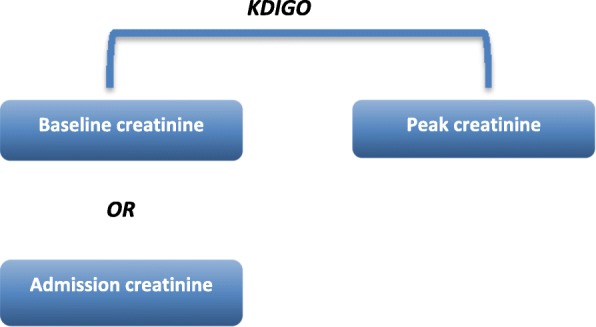
Flowchart of KDIGO Classification for AKI definition and Mortality Assessment. Baseline creatinine: previous value within the last 3 months of hospital admission. Peak creatinine: highest value within the first 7 days of hospital stay

All hospitalizations from the Emergency Department of the São Paulo Hospital, a university hospital linked to the Federal University of São Paulo, Brazil, between March 2015 and February 2017, were retrospectively evaluated. The inclusion criteria were a clinical diagnosis of hepatic cirrhosis, age above 18 years and a minimum hospital stay of 48 h. Exclusion criteria included renal and/or hepatic transplant patients, pregnant women and patients with chronic kidney disease who previously underwent dialysis. For those who were admitted to the emergency department more than one time within the specified period above, we only included data from the first admission. For the characterization and evaluation (incidence and mortality outcome) of AKI, the KDIGO 2012 criteria were applied, comparing peak creatinine and baseline creatinine (Fig. 1). Baseline creatinine was defined as the one prior to admission up to 3 months before hospitalization, which was obtained from the electronic medical record, or, in its absence, was considered to be the admission value, according to the guidance of the International Club of Ascites [14]. According to the common and widespread use in the literature, the final stage of AKI was defined by peak creatinine (the highest value obtained during the first 7 days of hospital admission) [2, 14, 15]. Diuresis data were not considered because of the difficulty of measuring diuresis in the emergency department, resulting in the absence of these data for most patients, and because it is not a very accurate measurement to evaluate kidney function in cirrhotic patients [14, 16, 17].

**Table 1:** Demographic, clinical and laboratory data among survivors and non-survivors

	Total	Survivors	Non-survivors	*p*
Patients, %	258	185 (71.7)	73 (28.4)	
Age, years (median, IQR)	59 (52;65)	57.9 (12.2)	58.7 (12.2)	0.681
Gender, male / female (%)	185 (71.7) / 73 (28.3)	130 (70.3) / 55 (75.3)	55 (29.7) / 18 (24.7)	0.447
Etiology, *n* (%)				
Alcohol	125 (48.4)	93 (50.3)	32 (43.8)	0.407
Viral	100 (38.8)	68 (36.8)	32 (43.8)	0.322
Non-viral and Non-alcohol	54 (20.9)	40 (21.6)	14 (19.2)	0.736
Days of hospitalization (median, IQR)	7 (3;13)	6 (3;11)	12 (6;23)	< 0.001
ICU admission (%)	78 (30.2)	35 (18.9)	43 (58.9)	< 0.001
admission MELD score (median, IQR)	18 (14;23)	17 (13;22)	19 (16;26)	0.002
admission APACHE II (median, IQR)	16 (12;22)	15 (12;20)	18 (14;23)	0.015
admission MAP (mmHg), (median, IQR)	89 (75;100)	91 (74;102)	84 (76;95)	0.036
Comorbidities, *n* (%)				
Hypertension	114 (44.2)	88 (47.6)	26 (35.6)	0.095
Diabetes	82 (31.8)	67 (36.2)	15 (20.5)	0.017
Smoking (present or past)	69 (26.7)	46 (24.9)	23 (31.5)	0.279
Cancer	57 (22.1)	32 (17.3)	25 (34.2)	0.004
Hepatocarcinoma	41 (70.7)	24 (72.7)	17 (68)	0.775
Non-hepatocarcinoma	17 (29.3)	9 (27.3)	8 (32)	
Heart failure	26 (10.1)	20 (10.8)	6 (8.2)	0.649
Cause of hospitalization, n (%)				
Infection	138 (53.5)	92 (66.7)	46 (33.3)	0.071
Non-infection	120 (46.5)	93 (77.5)	27 (22.5)	
AKI, *n* (%)				
Yes	139 (53.9)	80 (57.6)	59 (42.4)	< 0.001
No	119 (46.1)	105 (82.2)	14 (11.8)	
Progression of AKI, *n* (%)				
Yes	39 (27.9)	16 (41)	23 (59)	< 0.001
No	101 (72.1)	77 (76.2)	24 (23.8)	
Baseline eGFR (ml/min/1.73m^2^) (median, IQR)	57 (35;80)	59 (37;82)	52 (31;72)	0.055
Laboratory				
Hemoglobin, g/dL (mean, SD)	11.2 (2.75)	11.2 (2.7)	11.1 (2.8)	0.894
Albumin, g/dL (mean, SD)	2.9 (0.6)	3 (0.6)	2.7 (0.6)	0.008
Leucocytes, 1000/uL (median, IQR)	8.8 (5.7;12.4)	8.1 (5.4;11.0)	10.7 (7.3;14.1)	< 0001
Platelets, 1000/uL (median, IQR)	126 (80;186)	122 (74;180)	143 (94;195)	0.158
INR (median, IQR)	1.4 (1.2;1.6)	1.4 (1.2;1.6)	1.5 (1.3;1.7)	0.022
Total Bilirrubin, mg/dL (median, IQR)	2.2 (1.0;4.7)	1.7 (0.8;3.5)	2.9 (1.2;7.6)	0.002
Baseline Creatinine, mg/dL (median, IQR)	1.04 (0.80; 1.58)	1.02 (0.76;1.50)	1.10 (0.87;1.74)	0.054
Admission Creatinine, mg/dL (median,IQR)	1.40 (0.85; 2.34)	1.24 (0.80; 2.31)	1.61 (0.96; 2.50)	0.086
Peak Creatinine, mg/dL (median, IQR)	1.9 (1.01; 2.98)	1.47 (0.92; 2.48)	2.86 (1.93; 4.10)	< 0.001
Sodium mEq/L (median, IQR)	136 (132; 139)	137 (133;139)	136 (132;139)	0.197
Urea, mg/dL (median, IQR)	61 (34;99)	52 (33;89)	76 (38;104)	0.022

*AKI* Acute Kidney Injury. *MAP* Mean Arterial Pressure. *eGFR* Estimated Glomerular Filtration Rate. MELD and APACHE II were obtained from admission data

**Fig. 2:**
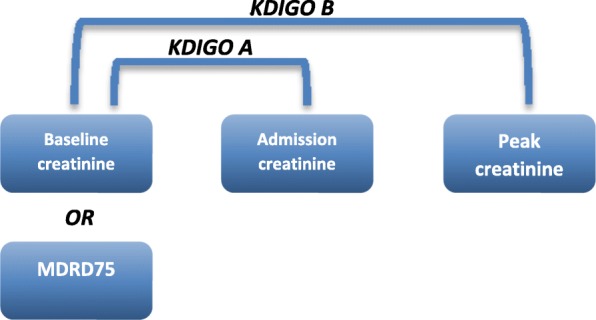
Flowchart of KDIGO Classification for AKI Progression. Baseline creatinine: previous value within the last 3 months of hospital admission. MDRD 75: Calculated creatinine value considering an eGFR of 75 ml/min/1.73m^2^ using the MDRD formula. Peak creatinine: highest value within the first 7 days of hospital stay. Progressors defined if KDIGO B > KDIGO A

For the analysis of the progression of AKI from admission to the first 7 days and its correlation with mortality, we performed a second classification of AKI according to the KDIGO criteria, comparing admission creatinine to the previous creatinine of hospitalization (baseline creatinine). Because we had no previous creatinine value for 109 patients (42% of total), we used the back calculation of creatinine considering a MDRD of 75 ml/min/1.73 m^2^ (MDRD-75) for these patients [16]. Both classifications were compared, admission and peak, in order to analyze the progression of AKI [18]. Progression of AKI was defined as the increase from lower stages of AKI to higher stages, such as AKI stage 1 to stage 2 or 3 or from stage 2 to stage 3 during the first week or until discharge [13] (Fig. 2).

**Table 2:** Demographic, clinical and laboratory data among AKI and non-AKI groups

	Total	Non-AKI	AKI	*p*
Patients, %	258	119 (46.1)	139 (53.9)	
Age, years (median, IQR)	59 (52;65)	59 (52;66)	60 (53;65)	0.630
Gender, male / female (%)	185 (71.7) / 73 (28.3)	83 (69.7) / 36 (30.3)	102 (73.4) / 37 (26.6)	0.580
Etiology, n (%)				
Alcohol	125 (48.4)	55 (46.2)	70 (50.4)	0.534
Viral	100 (38.8)	41 (34.5)	59 (42.4)	0.202
Non-viral and Non-alcohol	54 (20.9)	30 (25.2)	24 (17.3)	0.127
Days of hospitalization (median, IQR)	7 (3;13)			
ICU admission (%)	78 (30.2)	19 (16)	59 (42.4)	< 0.001
admission MELD score (median, IQR)	18 (14;23)	16 (11;19)	19 (16;25)	< 0.001
admission APACHE II (median, IQR)	16 (12;22)	14 (11;18)	19 (14;23)	< 0.001
admission MAP (mmHg), (median, IQR)	89 (75;100)	89 (76;100)	87 (74;100)	0.679
Comorbidities, *n* (%)				
Hypertension	114 (44.2)	51 (42.9)	63 (45.3)	0.708
Diabetes	82 (31.8)	36 (30.3)	46 (33.1)	0.688
Smoking (present or past)	69 (26.7)	30 (25.2)	39 (28.1)	0.673
Cancer	57 (22.1)	26 (21.8)	31 (22.3)	1
Hepatocarcinoma	41 (70.7)	19 (70.4)	22 (71)	1
Non-hepatocarcinoma	17 (29.3)	8 (29.6)	9 (29)	
Heart failure	26 (10.1)	11 (9.2)	15 (10.8)	0.836
Cause of hospitalization, *n* (%)				
Infection	138 (53.5)	52 (37.7)	86 (62.3)	0.004
Non-infection	120 (46.5)	67 (55.8)	53 (44.2)	
Baseline eGFR (ml/min/1.73m^2^) (median, IQR)	57 (35;80)	66 (35;89)	56 (35;73)	0.073
Laboratory				
Hemoglobin, g/dL (mean, SD)	11.2 (2.75)	11.3 (2.72)	11.1 (2.78)	0.493
Albumin, g/dL (mean, SD)	2.9 (0.6)	2.9 (0.6)	2.8 (0.6)	0.070
Leucocytes, 1000/uL (median, IQR)	8.8 (5.7;12.4)	7.7 (5.2;11)	10 (6.7;13.5)	0.001
Platelets, 1000/uL (median, IQR)	126 (80;186)	108.5 (70.5;174.5)	137 (88;192)	0.036
INR (median, IQR)	1.4 (1.2;1.6)	1.4 (1.2;1.6)	1.5 (1.3;1.7)	0.009
Total Bilirrubin, mg/dL (median, IQR)	2.2 (1.0;4.7)	2 (0.8;4)	2 (1;4.6)	0.370
Baseline Creatinine, mg/dL (median, IQR)	1.04 (0.80; 1.58)	0.96 (0.70;1.56)	1.05 (0.85;1.59)	0.081
Admission Creatinine, mg/dL (median,IQR)	1.40 (0.85; 2.34)	0.98 (0.72;1.60)	1.80 (1.13;2.77)	< 0.001
Peak Creatinine, mg/dL (median, IQR)	1.9 (1.01; 2.98)	0.99 (0.75;1.56)	2.55 (1.93;3.85)	< 0.001
Sodium (median, IQR)	136 (132; 139)	137 (133;140)	136 (132;139)	0.078
Urea, mg/dL (median, IQR)	61 (34;99)	39 (27;70)	78 (46;108)	< 0.001

*AKI* Acute Kidney Injury. *MAP* Mean Arterial Pressure. *eGFR* Estimated Glomerular Filtration Rate. MELD and APACHE II were obtained from admission data

Statistical analysis was performed with SPSS statistics software version 23.0 for Windows. Quantitative variables were represented by mean and standard deviation if the distribution was normal (Kolmogorov-Smirnov test) and were compared by Student’s T test. When a nonnormal distribution was characterized, median and interquartile range values were expressed, and the comparison was tested by the Wilcoxon test. Categorical variables were compared by the χ^2^ test (or Fischer exact test, when applicable). Possible risk factors for mortality that were identified in the univariate analysis with *p* value < 0.1 were included in models of logistic regression analysis. The data were presented as odds ratios and 95% confidence intervals. Values of *p* < 0.05 (2-tailed) were considered statistically significant.

Regarding the variables with missing data, we did not have previous creatinine values of 109 patients, as previously mentioned, and we did not have albumin values of 31 patients (12%). For the missing albumin data, we used the multiple imputation method to perform the necessary analysis.

This study was approved by the Ethics in Research Committee of the Federal University of São Paulo, and formal informed consent was waived because of the observational nature of the study.

## Results

**Fig. 3:**
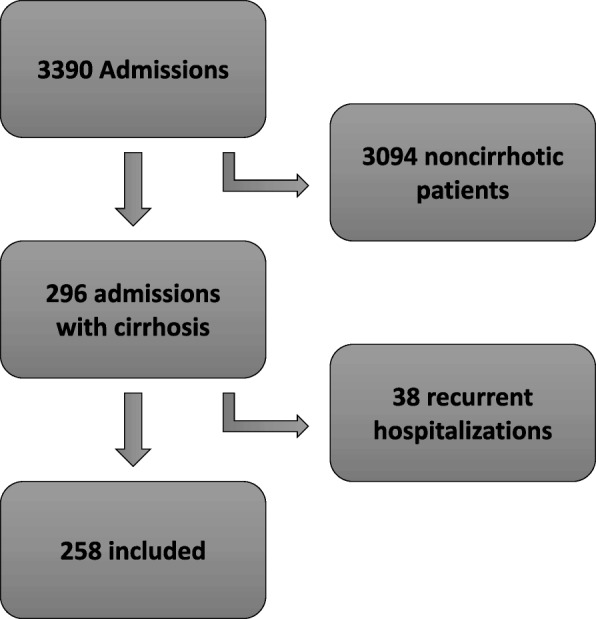
Derivation of the study cohort

The population studied included 258 patients (Fig. 3). From 3390 hospitalizations at the University Hospital Emergency Department, 3094 were noncirrhotic patients. Among the remaining 296 cirrhotic patients, 38 had recurrent hospitalizations, and only the first admission was considered for statistical analysis.

Clinical, laboratory, and demographic characteristics of patients included in the study are presented in Tables 1 and 2. These tables include the main comorbidities of patients in descending order of prevalence, mean length of hospital stay, ICU admission rate, APACHE II score from admission, MELD score from admission and admission mean arterial pressure (MAP). Regarding the etiology of cirrhosis, it was classified in three groups (viral, alcoholic and nonviral non-alcoholic), according to the two main causes of cirrhosis worldwide. The reason for admission to the emergency room was similarly divided into two groups, considering both infectious and noninfectious causes.

The overall mortality rate was 28.4%, with no significant difference in relation to the mean age (*p* = 0.681), sex (*p* = 0.447), the etiology of cirrhosis (alcoholic *p* = 0.407, viral *p* = 0.322, nonviral and non-alcoholic *p* = 0.736) or hospitalization due to infectious causes (*p* = 0.071). However, the nonsurvivor group had the highest median APACHE II admission (18 vs 15 *p* = 0.015), the highest median MELD score (19 vs 17 *p* = 0.002) and the highest ICU admission rate (58.9% vs 18.9% *p* = < 0.01). Among the comorbidities presented, patients with cancer presented higher mortality when compared to noncancer patients (34.2% vs 17.3% *p* = 0.004), but this difference was not statistically significant when the primary site was evaluated as hepatocarcinoma (*p* = 0.775) versus nonhepatocarcinoma tumors.

In multivariate analysis, variables with *p* < 0.1 according to univariate analysis were included in the model. Independent risk factors for in-hospital mortality are shown in Table 3.

When the criteria for AKI were applied for evaluation related to the first 7 days, 139 (53.9%) patients presented AKI: 55 (39.5%) patients with KDIGO 1, 35 (25.1%) with KDIGO 2 and 49 (35.2%) with KDIGO 3. Of the 49 patients with KDIGO 3, 18 (36.7%) required dialysis treatment. Among the patients with AKI, a higher admission rate in the ICU (42.4% vs 16%), higher APACHE II value (19 vs 14), higher MELD score (19 vs 16) and a higher incidence of infectious causes (62.3% vs. 37.7%) when compared to the non-AKI group were observed. There was no difference in relation to the etiology of cirrhosis or the presence of comorbidities between the groups. The overall mortality of the AKI group was 42.4%, and it was 11.8% in the non-AKI group.

**Fig. 4:**
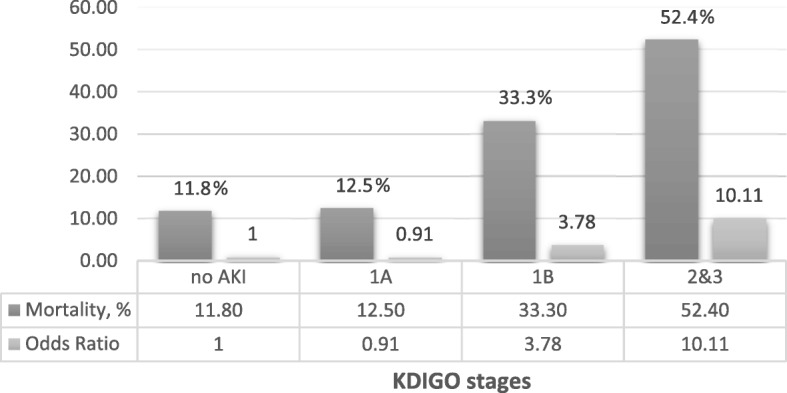
Logistic regression considering the groups with statistical difference between each other (1a, 1b, 2 and 3). Adjusted for age, sex, hypertension, diabetes, APACHE II score and MELD score. No AKI is the reference group (Odds Ratio = 1). AKI – Acute Kidney Injury. KDIGO stages and Mortality Analysis

The AKI subgroups of the KDIGO classification and the relationship with mortality are presented in Fig. 4 and Table 4. Within the KDIGO 1 group, 28 fulfill only elevation in creatinine ≥0.3 mg/dL in 48 h; the other 27 patients matched the criteria of elevation of 1.5× baseline creatinine at 7 days. Among these patients, 16 (29.1%) were KDIGO 1a and 39 (70.9%) KDIGO 1B. Differences in mortality with statistical significance were observed for the groups 2 and 3 when compared to group without AKI. In the analysis of KDIGO 1 subgroups, mortality was 12.5% and 33.3% for 1a and 1b, respectively, with significance only for subgroup 1b. There was no significant difference in mortality between the KDIGO 2 and 3 groups. The final model was adjusted for age, sex, hypertension, diabetes, APACHE II and MELD scores.

Logistic regression methods are shown in 2 models to mitigate collinearity between AKI admission diagnosis and AKI progression status (Table 3) - MODEL 1 consider admission criteria of AKI plus all covariates that presents with *p* < 0.1 in univariate analysis. MODEL 2 shows criteria of Progression of AKI plus all covariates that present with p < 0.1 in univariate analysis. In model 1, admission criteria of AKI, cancer diagnosis, length of stay in hospital, and leukocyte count (per 103 increase) are shown as risk factors for mortality. About model 2, the risk factors for mortality present as Progression of AKI, cancer diagnosis, Age (per year), MELD score at admission and length of stay in hospital.

**Table 3:** Logistic Regression – Risk Factors for In-Hospital Mortality

Variable	OR	95% CI	*p*
MODEL 1			
AKI	4.66	1.94–11.17	< 0.001
Cancer	3.94	1.74–8.89	< 0.001
Length of stay in hospital	1.07	1.03–1.10	< 0.001
Leukocytes	1.08	1.01–1.15	0.016
Diabetes	0.31	0.11–0.80	0.016
MAP	0.98	0.96–1.00	0.126
Age	1.02	0.98–1.05	0.195
MELD	1.04	0.97–1.11	0.247
APACHE II	0.97	0.88–1.05	0.427
Suspected infection	0.76	0.35–1.63	0.483
Baseline eGFR	1.07	0.77–1.47	0.694
Male Sex	1.16	0.51–2.59	0.726
Hypertension	0.89	0.37–2.09	0.788
Urea	0.99	0.98–1.01	0.868
MODEL 2			
Progression of AKI	12.05	3.29–44.07	< 0.001
Cancer	10.27	2.73–38.47	< 0.001
Age	1.08	1.02–1.14	0.006
MELD	1.15	1.03–1.26	0.006
Length of stay in hospital	1.08	1,02 - 1,13	0.007
Urea	1.01	0.99–1.02	0.073
MAP	0.98	0.95–1.00	0.090
APACHE II	0.93	0.82–1.05	0.240
Baseline eGFR	0.81	0.55–1.17	0.273
Hypertension	0.53	0.14–1.87	0.322
Leukocytes	1.04	0.95–1.13	0.367
Male Sex	1.26	0.38–4.10	0.702
Diabetes	0.89	0.26–2.99	0.852
Suspected infection	1.09	0.34–3.46	0.882

We considered the following units: per day for Length of stay in hospital, per year for Age, per 1 mg/dL for Urea, per 1 mmHg for MAP, per 1 ml/min/1.73m^2^ for eGFR, per 1 × 10^3^/μL. *AKI* Acute Kidney Injury. *MAP* Mean Arterial Pressure. *eGFR* Estimated Glomerular Filtration Rate. MELD and APACHE II were obtained from admission data

Finally, the progression of AKI occurred in 39 patients (27.9% of those with AKI). In those who progressed, mortality was 59% and was 23.8% in the nonprogression group (*p* < 0.001) (Table 4).

**Table 4:** AKI Stages and Mortality Outcome

Stages of AKI	Total	Mortality, %	Unadjusted			Adjusted^b^		
			OR	IC 95%	*p*	OR	IC 95%	*p*
no AKI^a^	119	11.8	1			1		
AKI	139	42.2						
KDIGO 1	55	27.3	2.81	1.24–6.35	0.013			
1a	16	12.5	1.07	0.22–5.21	0.932	0.91	0.18–4.60	0.916
1b	39	33.3	3.75	1.57–8.93	0.003	3.78	1.47–9.70	0.006
KDIGO 2 and 3	84	52.4				10.11	4.36–23.43	< 0.001
KDIGO 2	35	40.0	5.00	2.08–12.01	< 0.001			
KDIGO 3	49	61.2	11.84	5.31–26.37	< 0.001			

^**a**^Reference group. ^b^Adjusted for age, sex, hypertension, diabetes, APACHE II score and MELD score. AKI, acute kidney injury

## Discussion

This study presented the evaluation of 258 cirrhotic patients admitted to an emergency unit. In general, the mortality rate (28.4%) was similar to that reported for similar cohorts [1, 6–8, 12, 15]. The most common etiology was similar to that of national and international cohorts [17], with alcoholic cirrhosis being the most common followed by viral etiology (mainly secondary to hepatitis C). Due to the unavailability of the level of ascites in electronic records of some patients and the heterogeneous classification of hepatic encephalopathy, a Child-Pugh-Turcotte classification was not possible, but we inferred from clinical and laboratory data that the majority of patients would be classified as having at least Child B liver cirrhosis with a mortality similar to that reported in the literature [8].

This investigation has shown that patients with AKI in progression have an increasing mortality according to the classification of renal impairment (Fig. 4), which reinforces the need for a temporal and progressive evaluation of renal function. In addition, in this population, the presence of progression of AKI and peak creatinine in the first 7 days of admission presented better performance when compared to the isolated creatinine value of entry, regarding mortality. Surprisingly, the baseline eGFR prior to hospital admission did not present as a risk factor for mortality or acute kidney injury during hospitalization, as widely established in the literature of noncirrhotic patients [18]; however, there are already clinical trials in cirrhotic patients with results similar to our study [6].

Multivariate analysis showed that both AKI diagnosis at admission and AKI Progression criteria are major risk factors for mortality; this information suggests that cirrhotic patients with less severe AKI (KDIGO 1) need a special approach and structured care. Surprisingly, the APACHE II score lost its power of discrimination on both models in multivariate analysis. Another study found similar results [19] and presented a possible explanation that the Apache II score lacks a liver-specific prognostic factor in its calculator, so it is probable that the Apache II score will lose its strength when compared with another specific variables. In model 1, the MELD score lost its ability to predict mortality risk probably because of collinearity between the admission creatinine and calculated admission MELD.

As shown in Table 4, the presence of AKI KDIGO 1b was shown to be a risk factor for mortality, different from the presence of AKI KDIGO 1a. This fact corroborates the subclassification proposed by the International Club of Ascites [13], which reinforces that small elevations in the value of creatinine, especially when they exceed 1.50 mg / dL, have a great impact on the morbidity and mortality of patients with cirrhosis.

No significant difference in mortality was found between the stages without AKI and KDIGO 1a, and the KDIGO stages 2 and 3. It is possible to identify three major progressive mortality groups: KDIGO 1a, KDIGO 1b and KDIGO 2/3, with the prognosis of the first group comparable to the absence of AKI, reinforcing previous data [15].

There are several limitations of our study. Although the present investigation was based on a retrospective cohort, it presents an analysis of a considerably large number of patients, regarding this specific population of cirrhotic patients in an emergency department, which ought to be considered innovative in the literature. Due to missing clinical data, we were not able to achieve a Child-Pugh classification or include the etiology of AKI. Since we designed this study to assess only the first week in the hospital, we did not evaluate the occurrence of late AKI (after 7 days). Another limitation is the lack of data on AKI duration, since patients who improve within 48 h may represent a phenotype of AKI with a better prognosis. Final limitation is the possible misclassification of chronic kidney disease as AKI when analyzing progression of AKI, since those patients without previous creatinine value were considered as having a eGFR of 75 ml/min/1.73m^2^.

## Conclusions

According to the data presented above, a single measure of creatinine is not enough in this population, as this paper reinforces the need for meticulous follow-up of the renal function of patients with hepatic cirrhosis hospitalized in an emergency unit, in view of the correlation of renal function with clinical outcomes. In addition, in agreement with the current literature, the data reinforce the need for subclassification of KDIGO 1 in cirrhotic patients, demonstrating that patients with acute renal injury and creatinine greater than 1.5 mg/dL present a worse clinical outcome.

## Abbreviations

AKI: Acute Kidney Injury
eGFR: Estimated Glomerular Filtration Rate
ICU: Intensive Care Unit
KDIGO: Kidney Disease: Improving Global Outcomes
MAP: Mean Arterial Pressure
MDRD: Modification of Diet in Renal Disease Formula for eGFR
